# Disseminated Histoplasmosis with Underlying Sarcoidosis-Rheumatoid Arthritis Overlap Syndrome: An Example of Diagnostic Test Threshold of Detection Affecting Test Results and Patient Care

**DOI:** 10.1155/2022/8334083

**Published:** 2022-07-14

**Authors:** Daniel Pankratz, Jordan Tichenor, Fernando Merino, Nathan C. Bahr

**Affiliations:** ^1^School of Medicine, University of Kansas Medical Center, Kansas, KS, USA; ^2^Division of Infectious Diseases, Department of Internal Medicine, University of Kansas Medical Center, Kansas, KS, USA

## Abstract

Histoplasmosis is common in many parts of the world but with areas of hyperendemicity. Disseminated histoplasmosis is the deadliest form of histoplasmosis and is most common among immunocompromised patients. Timely diagnosis is crucial to improve outcomes. We describe a patient on azathioprine and rituximab with disseminated histoplasmosis in which diagnosis was delayed in part because of negative early *Histoplasma* antigen testing, which was positive later in the course. This case serves as an example of the concept of “threshold of detection” in which a certain concentration of a microbe must be present for it to be detected by a diagnostic test that focuses on detection of a microbe or its components. This concept applies to many tests used to diagnose infectious diseases.

## 1. Case Report

A 40-year-old female presented to an emergency department with cough, body aches, headache, fevers up to 39.4°C, and shortness of breath for one week (day zero). The patient had a 12-year history of rheumatoid arthritis (RA) and a five-year history of sarcoidosis, thereafter designated RA-sarcoid overlap syndrome. Her therapy was azathioprine daily and rituximab infusions every four months, with her last infusion three months prior to presentation. The complete blood count with differential, c-reactive protein, and procalcitonin were drawn (Table 1), and numerous tests including COVID-19 PCR, rapid influenza A/B antigens, urinalysis, and blood cultures were sent and were found to be negative or within normal limits. She was found to have an elevated D-dimer and underwent a chest computed tomography angiography (CTA). This did not show evidence of pulmonary embolism but did reveal scattered ground glass opacities in both lungs. Her symptoms and imaging studies were thought to be consistent with an “atypical” bacterial pneumonia, and she was subsequently discharged from the emergency department on doxycycline 100 mg twice daily for one week.

Three days later (day + 3), the patient continued to have fevers and chills. Levofloxacin was added to her regimen. The patient eventually required hospital admission for persistent fever. On admission, she was started on levofloxacin, ertapenem, and vancomycin. An extensive workup was completed during her admission, which included two sets of blood cultures, lactate dehydrogenase (LDH), serum cryptococcal antigen, urine and serum *Histoplasma* antigens, and 1,3 beta D glucan assay (Table 1) which were all negative or within normal limits, except for LDH which was slightly elevated. A two-view chest X-ray was without any lobar consolidations or infiltrates. Pulmonology was consulted and performed a bronchoscopy. Cytology from bronchoalveolar lavage (BAL) returned negative for malignancy and Grocott's methamine silver stain was negative for fungi. BAL culture grew *Streptococcus parasanguinous* and *Rothia dentocaiosa*. She was discharged on levofloxacin for five additional days.

**Figure 1:**
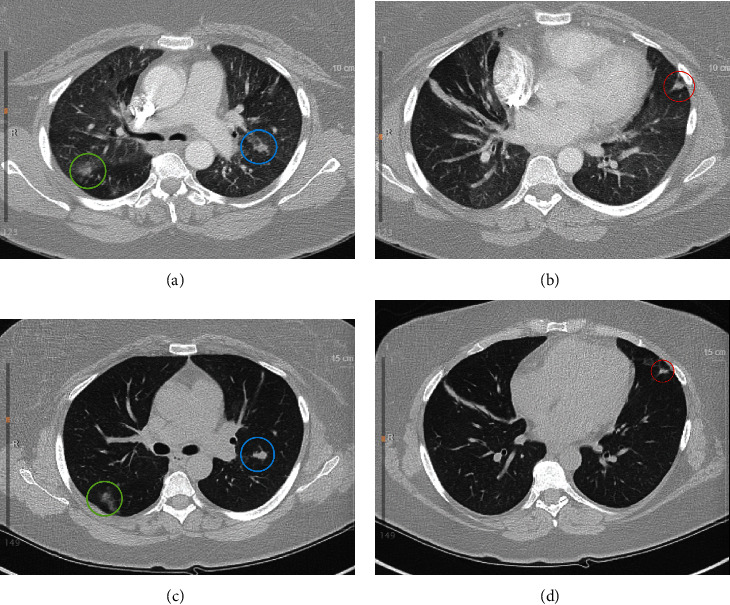
CT chest images from the initial and subsequent hospital presentations. (a) and (b) show CT scan without contrast performed during the patient's initial presentations demonstrating pulmonary nodules in the left and right upper lobes with blue and green circles, respectively, and a left pulmonary nodule at the base of the left lung with red circle. (c) and (d) show CT scan without contrast performed one month after the patient's initial consultation demonstrating similar pulmonary nodules to her preliminary visit with nodules identified by the same color circle as in (a) and (b).

Approximately four weeks after the onset of her initial symptoms (day + 30), the patient reported continued fevers ranging from 38.9–39.4°C. At this time, it was thought that her symptoms could be due to inflammatory changes related to COVID-19 despite numerous negative COVID-19 PCR tests. A COVID-19 antibody test was ordered and she was started on 6 mg of dexamethasone and amoxicillin daily. The following day, viral cultures from her BAL returned positive for cytomegalovirus (CMV). With positive CMV respiratory viral cultures, recurrent fevers, and ground glass opacities on the CT from her initial presentation, (Figures 1(a) and 1(b)), CMV pneumonitis was suspected and she was then started on valganciclovir 900 mg twice daily. The patient reported improvement of her symptoms one week after starting valganciclovir although she continued to suffer from dyspnea, cough, and fever. Her COVID-19 antibody test returned negative and dexamethasone and amoxicillin were discontinued.

Five weeks after her initial presentation (day + 35), the patient was admitted to our medical center. At time of admission, the patient also reported headache sinus congestion and drainage, increased cough and dyspnea on exertion, poor appetite, weight loss, and vague rib pain. She was started on IV ganciclovir for her suspected CMV pneumonitis. Her fever continued for four days (maximum 39.4°C). She had mild elevated aspartate and alanine transaminase and alkaline phosphatase levels, anemia and leukopenia (Table 1). Additional negative/normal laboratory tests included blood cultures, COVID-19 PCR, a multiplex respiratory viral panel from a nasopharyngeal swab, HIV antibody/antigen testing, *Aspergillus* galactomannan antigen, *Brucella* IgG/IgM, *Coxiella burnettii* phase 1 and phase 2 IgG and IgM, blood culture for acid fast bacilli, *Coccidiodes* IgG/IGM and urine antigen, *Cryptococcus* serum antigen, 1,3 beta *D* glucan, blood PCR for Epstein-Bar virus, and blood PCR for CMV. Lumbar puncture was also obtained which demonstrated a white blood cell count of 3 cells/*μ*L, glucose of 58 mg/dL, and protein of 38 mg/dL. Computed tomography (CT) of the chest showed innumerable small pulmonary nodules throughout both lungs, many demonstrating a peri lymphatic distribution (Figures 1(c) and 1(d)). The CT was comparable to a CT done during her hospitalization one month prior to it but, however, showed increased nodules when compared to CT taken from outside 6 months earlier. Lung biopsy was considered but ultimately deferred due to the absence of a safe target for the procedure.

**Figure 2:**
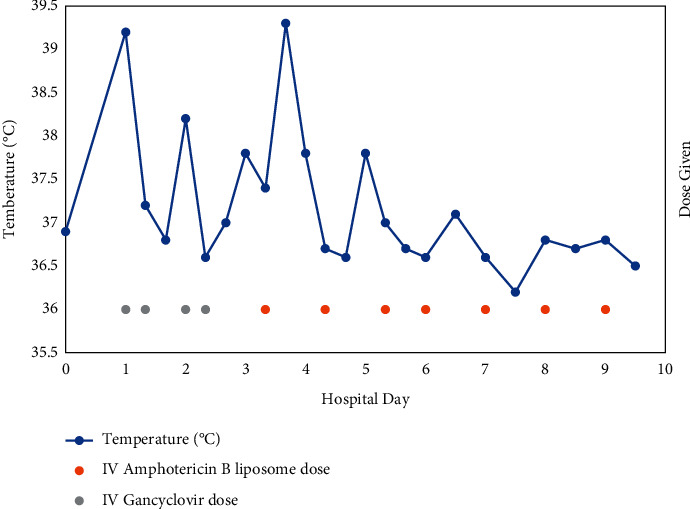
Temperature response to the initiation of antifungal therapy. Graph of the patient's temperature throughout until her final consultation to the hospital and diagnosis of disseminated histoplasmosis. This graph shows the patient's temperature throughout her final hospitalization. Each dose of IV ganciclovir and IV amphotericin are indicated by gray and orange dots, respectively.

With her history of treatment with oral valganciclovir for two weeks and IV ganciclovir during her admission with no improvement in her fevers, it was determined that CMV pneumonitis was unlikely to be the source of her fevers. Thus, her IV ganciclovir treatment was discontinued on hospital day three (day + 38). Later, on the same day, lab results obtained showed that a positive *Histoplasma* urine antigen of 6.56 ng/mL (results below 0.4 ng/mL but >0 reported as positive below the limit of quantification) and a positive *Histoplasma* serum antigen of 0.49 ng/mL. Given this result in the context of a compatible clinical picture, she was diagnosed with disseminated histoplasmosis, and treatment was started with liposomal amphotericin B at 3 mg/kg per day, followed by rapid resolution of her fever and symptoms (Figure 2). She continued daily infusions of liposomal amphotericin B for 14 days (end = day + 53).

Thereafter, amphotericin B was discontinued and the patient was transitioned to PO itraconazole 200 mg three times daily for three days followed by 200 mg twice daily. The patient was discharged with weekly therapeutic drug monitoring. Prior to discharge from our facility after completing her 14-day course of amphotericin, the fungal culture from her BAL during her initial hospitalization grew *Histoplasma capsulatum*. During a two-week followup after discharge (day+67), the patient reported moderate improvement of her dyspnea with activity and fevers remained absent. During her three-month follow-up (day + 143), she only had residual shortness of breath with activity. Azathioprine was restarted one month after discharge and rituximab was resumed six months after discharge. Itraconazole was planned for at least one year. She relocated and transferred health care to a local physician prior for completion of therapy.

## 2. Discussion

Disseminated histoplasmosis is typically an opportunistic infection seen in those who have an impaired immune system, particularly advanced HIV [1]. However, many rheumatologic and oncologic conditions require immune suppressive therapy, and *Histoplasma* should be considered a potential etiology in the proper clinical setting for such patients as well, especially in hyperendemic areas [2]. One estimate of the incidence of histoplasmosis in adults who are 65 and older is 6.1 cases per 100,000 person/year in the midwest United States compared to 3.5 cases per 100,000 person/year in the southern United States and 1.1 cases per 100,000 person/year in the northeast and western regions of the United States [3]. Importantly, while a higher index of suspicion is warranted in areas of higher incidence, it is now clear that histoplasmosis also occurs outside of traditional endemic areas. [2].

There are multiple reports of disseminated histoplasmosis seen as early as 1965 in settings where azathioprine was used [4–7]. Azathioprine is a common medication used in immunosuppression for the treatment of systemic autoimmune diseases. While the literature shows that azathioprine has been implicated in increasing susceptibility to opportunistic infections, *Histoplasma* infection is a rare complication [8].

This patient was not definitively proven to have disseminated histoplasmosis via lung or bone marrow biopsy as risks of the procedures were felt to outweigh potential benefits. However, her leukopenia, anemia, liver function test abnormalities, innumerable lung nodules, and presentation in the setting of a significantly elevated urine *Histoplasma* antigen test make dissemination likely.

A key point in this case was the delay in diagnosis from her first presentation to the treatment of her *Histoplasma* infection. There are multiple reasons for the delay. The patient's initial presentation was considered likely to be a more common bacterial infection. Subsequently when her symptoms continued, despite antibiotic treatment, she was suspected to have CMV pneumonitis. CMV testing on BAL is frequently positive without true CMV disease in the setting of immune suppression and lung disease [9,10]. In this case, CMV being considered as a probable diagnosis led to some delay in additional workup as well.

Prior to CMV being positive by BAL culture, histoplasmosis was considered but antigen testing was negative. Over a month later when she was rehospitalized, *Histoplasma* antigen testing was positive. Eventually her BAL culture (from her initial hospitalization) grew *Histoplasma capsulatum*, but this result was not available until after repeat antigen testing confirmed the diagnosis. This is a predictable scenario. Although culture is often considered a gold standard for histoplasmosis, results take 2–8 weeks, frequently too long to aid in diagnosis [11].

Relying on her antigen tests to rule out histoplasmosis early in her course also may have delayed treatment. Antigen testing in isolated pulmonary histoplasmosis is not sensitive enough to rule out the disease [12]. The same is true with disseminated histoplasmosis where the fungal burden is low. The concept of “threshold of detection” is important in this case. The patient may have had antigen in her blood and/or urine but not to the level required for detection by the available assays (the required concentration has not formally been published to our knowledge but the same commercial test was used, MiraVista labs). As her course continued and her infection worsened, it is likely that more *H capsulatum* in her body led to increasing antigen levels—allowing detection. In general, any test meant to detect a pathogen has an inherent threshold above which the pathogen may be detected; here, it is likely that the amount of *H. capsulatum* in the patient's body had not yet reached that limit when antigen testing was initially done.

## 3. Conclusion

This case underscores the importance of early diagnosis in cases of disseminated histoplasmosis. Disseminated histoplasmosis should be considered in any immunosuppressed patient who lives in or has traveled to an endemic region (including nontraditional endemic regions) with fever of unknown origin, particularly with pulmonary abnormalities. When considering histoplasmosis, one must be aware of the potential for false negative tests, particularly early in the course of disease. In persons who are highly suspected to have histoplasmosis, repeated antigen testing and alternative testing such as tissue culture and histopathology and/or empiric treatment should be considered. This case serves as a reminder of the imperfect nature of diagnostic testing which may delay time to treatment and result in poor outcomes.

## Figures and Tables

**Table 1:** Selected laboratory test results.

Component	Initial presentation (0+)	Day 5+	Five weeks after initial presentation (day 35+)^*∗*^	One month after initiation of therapy (day 67+)
Hemoglobin (gm/dL)	12.5	13.0	11.4	10.9
WBC (K/UL)	4.2	5.1	1.5	3.7
Neutrophil percent (%)	45	60.1	67	N/A
Lymphocyte percent (%)	31	23.3	26	N/A
Eosinophil percent (%)	7	6.1	3	N/A
C-reactive protein (mg/dL)	5.1	3.9	4.45	N/A
D-dimer (ug/mL)	1.38	N/A	N/A	N/A
Procalcitonin (ng/mL)	0.06	0.04	0.12	N/A
Histo urine Ag (ng/mL)	N/A	<0.4	6.56	N/A
Histo serum Ag (ng/mL)	N/A	<0.4	0.49	N/A
1,3 beta D glucan (pg/mL)	N/A	<31	<31	N/A
AST (U/L)	35	55	60	18
ALT (U/L)	37	83	62	17
ALP (U/L)	83	164	120	72
Lactate dehydrogenase (U/L)	N/A	265	N/A	N/A

^*∗*^Diagnosis of histoplasmosis.

## Data Availability

No data were used in this study.

## Abbreviations

WBC: white blood cells
N/A: not applicable
Ag: antigen
AST: aspartate aminotransferase
ALT: alanine aminotransferase
ALP: alkaline phosphatase
